# Early-stage multi-differentiated gastric carcinosarcoma and post-resection local recurrence: a case report

**DOI:** 10.1186/s13000-020-01037-4

**Published:** 2020-09-24

**Authors:** Akihiro Shioya, Nozomu Kurose, Kenichi Mizutani, Motona Kumagai, Ken Kawaura, Naohiko Nakamura, Takeo Kosaka, Nozomu Motono, Hidetaka Uramoto, Sohsuke Yamada

**Affiliations:** 1 Department of Pathology and Laboratory Medicine, Kanazawa Medical University, 1–1 Daigaku, Uchinada, Kahoku, Ishikawa 920–0293 Japan; 2 Department of Gastroenterological Endoscopy, Kanazawa Medical University, 1-1 Daigaku, Uchinada, Kahoku, Ishikawa 920-0293 Japan; 3 Department of Surgical Oncology, Kanazawa Medical University, 1-1 Daigaku, Uchinada, Kahoku, Ishikawa 920-0293 Japan; 4 Department of Thoracic Surgery, Kanazawa Medical University, 1-1 Daigaku, Uchinada, Kahoku, Ishikawa 920-0293 Japan

**Keywords:** Gastric carcinosarcoma, AFP-positive, Adenocarcinomatous, Case report, Metastasis, Recurrence, Gastric carcinoma

## Abstract

**Background:**

Carcinosarcoma is a rare neoplasm with a poor prognosis that is most often discovered at an advanced stage; a gastric carcinosarcoma is even rarer than carcinosarcomas originating in other organs, such as the uterus. We report our experience with an early-stage multi-differentiated gastric carcinosarcoma.

**Case presentation:**

A 68-year-old male patient presented with anemia, and his fecal occult blood test was positive. An endoscopic examination was conducted which revealed a hemorrhagic, irregular, protruding lesion in the stomach. The lesion was diagnosed as an adenocarcinoma by histopathological examination of the biopsy specimen, and a segmental gastrectomy was performed. A 41 × 29 × 18 mm^3^ protruding lesion was observed in the resection specimen, and histologically confirmed to be a gastric carcinosarcoma with mixed adenocarcinomatous and sarcomatous composition. Tumor invasion was limited to the submucosa. Besides the adenocarcinomatous portion, neuroendocrine differentiation and AFP-positive gastric carcinoma were present in the carcinomatous portion of the tumor; in the sarcomatous portion, chondrosarcomatous, leiomyosarcomatous, and rhabdomyosarcomatous components were observed in addition to the undifferentiated sarcomatous component. Furthermore, the tumor included SALL4-positive germ cell-like cells. Despite early-stage detection, the cancer recurred locally 14 months after tumor resection, which necessitated a total gastrectomy. At the 2-month follow-up after the total gastrectomy, the patient was alive. This patient had developed an esophageal squamous cell carcinoma and primary lung adenosquamous carcinoma, both of which were resected.

**Conclusions:**

Few cases of early-stage gastric carcinosarcoma have been reported, but there are no reports of recurrence to date. Local recurrence as in this patient, and even in early-stage cases, requires cautious surveillance to check for post-resection recurrence and metastasis. The etiopathogenesis of carcinosarcoma has not yet been elucidated; however, in the present case, despite the tumor’s relatively small size, it exhibited various types of differentiation in both the carcinomatous and sarcomatous components and a proliferative germ cell-like portion, which suggests that the monoclonal origin hypothesis may be a valid theory for the carcinosarcoma.

## Introduction

A carcinosarcoma is a malignant tumor with an admixture of carcinomatous and sarcomatous components, and it is a rare neoplasm regardless of the organ of origin. Typical sites of carcinosarcoma include the uterus, ovaries, breasts, esophagus, thyroid, lungs, larynx, and urinary tract [1, 2]. However, only a few cases of primary gastric carcinosarcoma have been reported; to date, less than 100 cases have been reported since Queckenstadt’s report in 1904 [3–5]. Kuroda et al. reported a mean patient age of 62.5 years and male predominance (male: female ratio of 5:2.2) with regard to gastric carcinosarcoma. The macroscopic tumor morphology is predominantly the protruding type, comprising large masses with a mean tumor diameter of 8.6 cm. Most cases of carcinosarcoma are discovered at an advanced stage, and many cases present with lymph node and distant metastasis [5]. Surgical resection is the commonest treatment for carcinosarcoma [1, 5]; however, the post-resection prognosis is poor, with a mean survival time of 7 to 10 months [5]. Histologically, the carcinomatous component is an adenocarcinoma in most patients, although a few reports exist on cases that include adenosquamous carcinoma [6] or adenocarcinoma with neuroendocrine differentiation [7]. The sarcomatous component is frequently an undifferentiated sarcoma, despite known cases with rhabdomyosarcomatous, leiomyosarcomatous, chondrosarcomatous, osteosarcomatous, and fibrosarcomatous differentiation; nonetheless, cases that include multi-type gastric ectopic components are relatively rare.

In this report, we present our clinical experience with gastric carcinosarcoma, wherein both the carcinomatous and the sarcomatous components showed various types of differentiation and recurred locally despite early-stage detection and excision when tumor invasion was limited to the submucosa.

## Case presentation

**Fig. 1:**
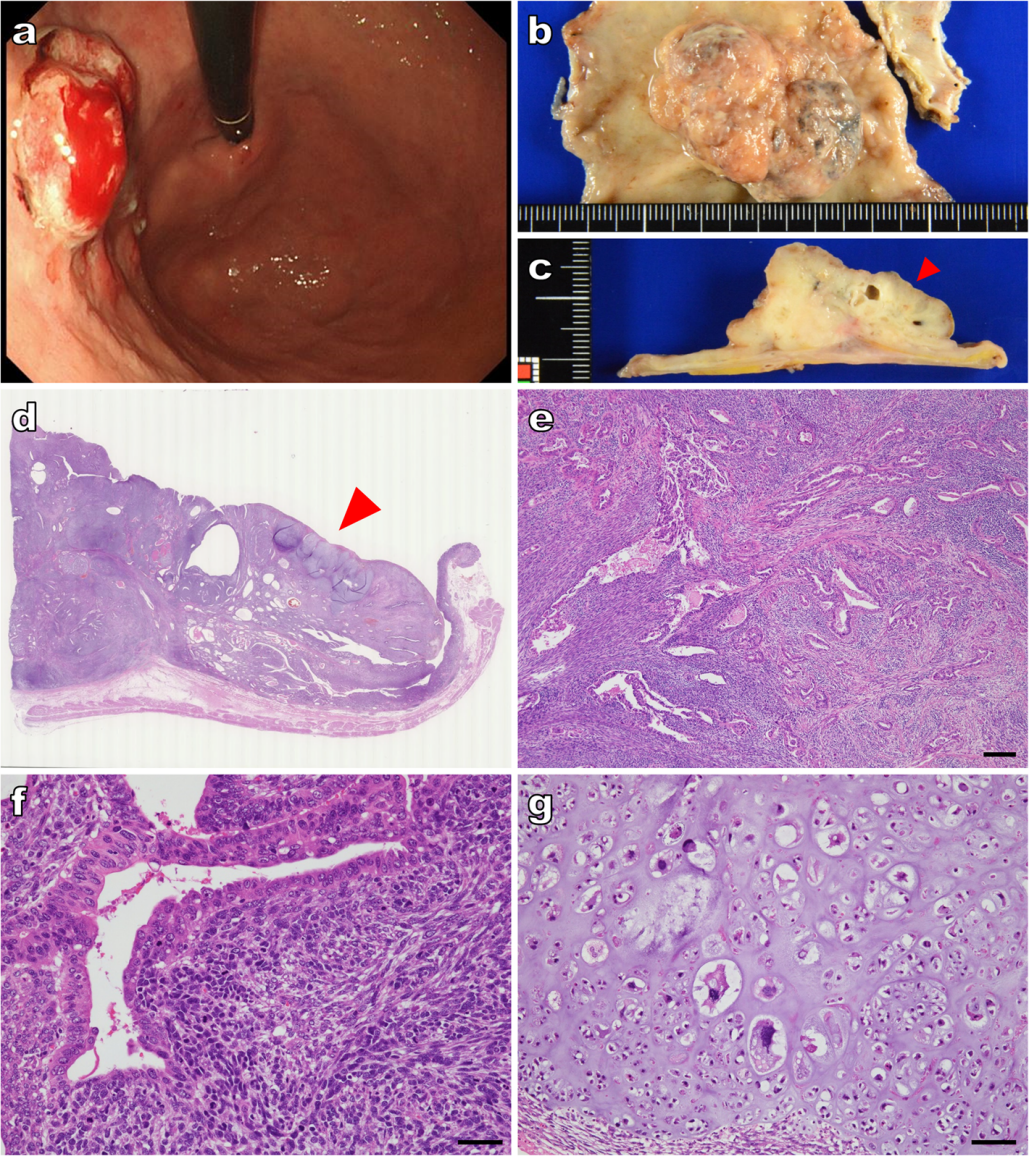
Endoscopic images showing the gastric mucosa at various levels of magnification. **a** Bleeding associated with an irregular, protruding lesion observed on the rear wall of the lesser curvature in the body of the stomach. **b** Segmental gastrectomy specimen. A 41 × 29 × 18 mm^3^ protruding lesion is observed. **c** The cross-section shows a grayish-white tumor with growth mainly on the mucous membranous side, partly composed of cystoid portions and a translucent cartilage-like matrix (indicated by the red arrow). **d** Low magnification (× 0.4) imaging indicating carcinosarcoma invasion limited to the submucosa. Cartilaginous tissue is visible indicated by the red arrow. **e** Magnified imaging (× 20 magnification) indicating an adenocarcinomatous component with tubular and papillary growth mixed with a sarcomatous component (scale bar 200 μm). **f** Higher magnification (× 200) imaging, indicating an acidophilic and cylindrical adenocarcinomatous component as well as a sarcomatous component with atypical spindle cell and atypical round cell proliferation with a high N/C ratio (scale bar 50 μm). **g** Magnified imaging (× 100) of a region showing chondrogenesis. Dyskaryosis is visible in chondrocyte-like cells (scale bar 100 μm)

**Fig. 2:**
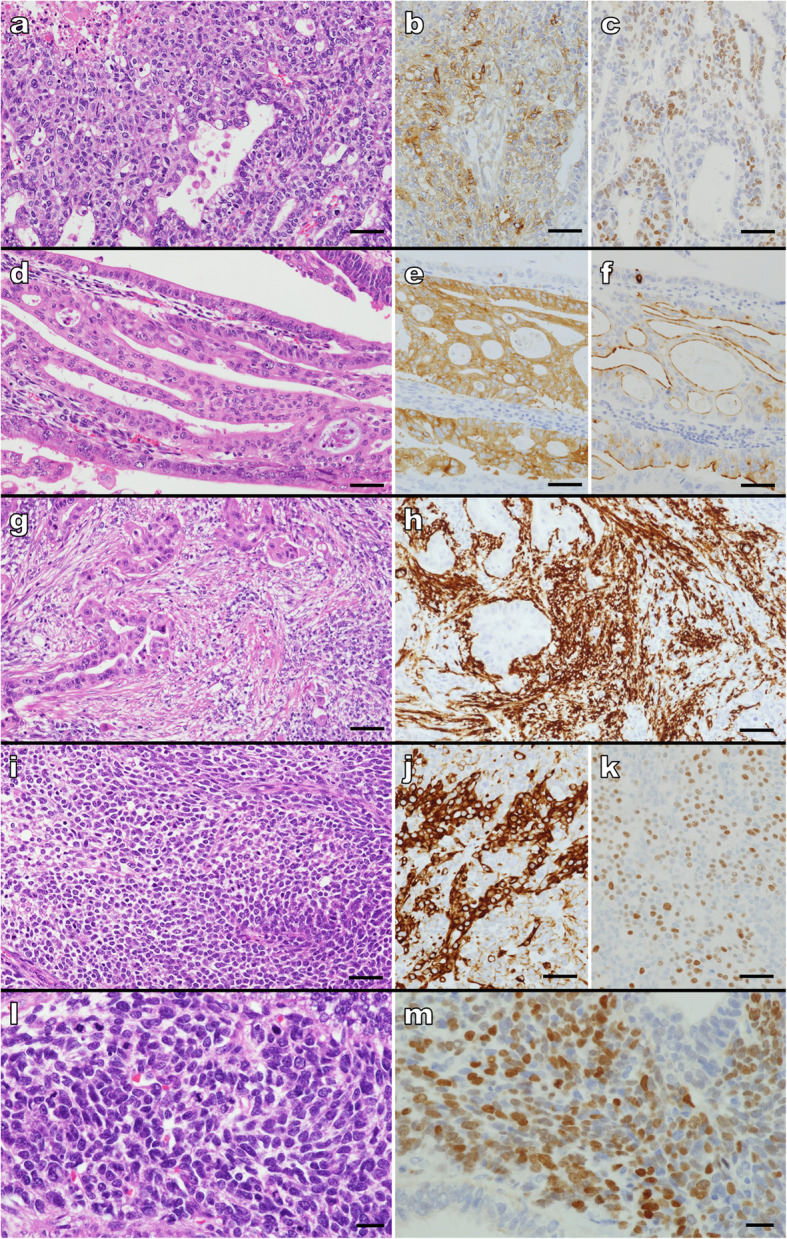
Immunohistochemistry (IHC) of the gastric carcinosarcoma showing H&E, AFP, and SALL4 immunostaining. **a** Tubular and solid growth patterns of adenocarcinoma with clear cytoplasm are visible; **b** The same area is positive for AFP by IHC; **c** Positivity for SALL4 staining is also shown; **d** indicates H&E staining showing a moderately differentiated adenocarcinomatous region with acidophilic cytoplasm; **e** this area indicates positivity of synaptophysin by IHC; **f** is also positive for chromogranin A; **g** is an H&E staining indicating proliferation of spindle cells with acidophilic cytoplasm; **h** this area is positive for SMA by IHC; **i** shows an H&E stained section indicating proliferation of atypical round cells; **j:** indicates positivity of desmin; and **k** MyoD1 by IHC; **l:** show a proliferative area of germ cell-like cells and proliferation of atypical “bare nuclei” cells is visible; **m** Immunohistochemically, the tumor is positive for SALL4. (scale bars in **a–k**: 50 μm and **i, m:** 20 μm)

A 68-year-old man with a history of hypertension, diabetes, and chronic kidney failure was undergoing treatment as an outpatient for angina pectoris and atrial fibrillation that was diagnosed 2 years prior to the carcinosarcoma. He reported a history of heavy smoking for approximately 40 years; moreover, his father had a history of lung cancer. The patient did not have any clinical symptoms of cancer, and no remarkable changes were noted on physical examination. However, a routine blood test done 3 months earlier indicated anemia (hemoglobin: 9.6 g/dL), and the patient’s stool sample tested positive for occult blood. We tested for tumor markers and found the squamous cell carcinoma (SCC) antigen was slightly elevated at 3.3 ng/mL, but the carcinoembryonic antigen (CEA) and CA 19–9 were within the reference range. An upper GI endoscopy showed a bleeding, irregular, protruding lesion located on the posterior wall of the lesser curvature within the body of the stomach (Fig. 1a); the lesion was biopsied and identified as a poorly differentiated adenocarcinoma. Moreover, at the lower part of the esophagus, there was a slightly concave lesion that was entirely separate from the gastric tumor. Biopsy specimens from the esophageal lesion indicated SCC. The patient was scheduled for surgical tumor excision. First, en bloc resection via endoscopic submucosal dissection was performed for the esophageal cancer, and the pathological diagnosis was well-differentiated SCC with negative margins and slight infiltration of the mucosal lamina propria. At a later date, segmental gastrectomy was performed for the gastric tumor. The proximal gastric surgical margin was confirmed to be negative by rapid assessment. The resected gastric tumor was subjected to histopathological examination. Macroscopically, the gastrectomy specimen had a protruding lesion measuring 41 × 29 × 18 mm^3^ (Fig. 1b). The cross-section showed a grayish-white tumor with growth mainly on the mucous membranous surface as well as areas with a cystic appearance and a translucent cartilage-like matrix in parts (Fig. 1c). Histologically, the tumor was a carcinosarcoma with mixed adenocarcinomatous and sarcomatous components (Fig. 1d,e). Tumor invasion was limited to the submucosa. The adenocarcinomatous component exhibited tubular, papillary, and, in some parts, solid growth patterns. The adenocarcinoma cells were acidophilic and cylindrical; however, some regions comprised adenocarcinoma cells with clear cytoplasm. The adenocarcinomatous component resulted in diffuse lymphatic and venous invasion. The sarcomatous portion showed proliferation of atypical spindle cells and atypical round cells with a high nucleus-to-cytoplasm (N/C) ratio (Fig. 1f). In the sarcomatous portion, some parts showed chondrogenesis, and dyskaryosis was observed in chondrocyte-like cells (Fig. 1g). Immunohistochemically, the adenocarcinomatous component with clear cytoplasm comprised areas with alpha-fetoprotein (AFP)- and Sal-like protein 4 (SALL4)-positive AFP-producing gastric carcinoma (Fig. 2a, b, c). Moreover, there were synaptophysin- and chromogranin A-positive adenocarcinomatous regions that showed neuroendocrine differentiation (Fig. 2d, e, f). The sarcomatous portion was predominantly composed of undifferentiated areas as indicated by unstained regions, but included smooth muscle actin-positive leiomyosarcomatous areas composed of spindle cells with acidophilic cytoplasm (Fig. 2g, h) as well as areas of desmin- and MyoD1-positive atypical round cells with rhabdomyosarcomatous differentiation (Fig. 2i, j, k). Furthermore, a proliferative focus with atypical “bare nucleus” cells that was partly composed of SALL4-positive germ cell-like cells did not indicate any specific differentiation in immunostaining (Fig. 2l, m). Histopathological analysis of the background stomach revealed chronic gastritis with intestinal metaplasia and negativity for *Helicobacter pylori*. There was no dysplasia around the gastric tumor.

**Fig. 3:**
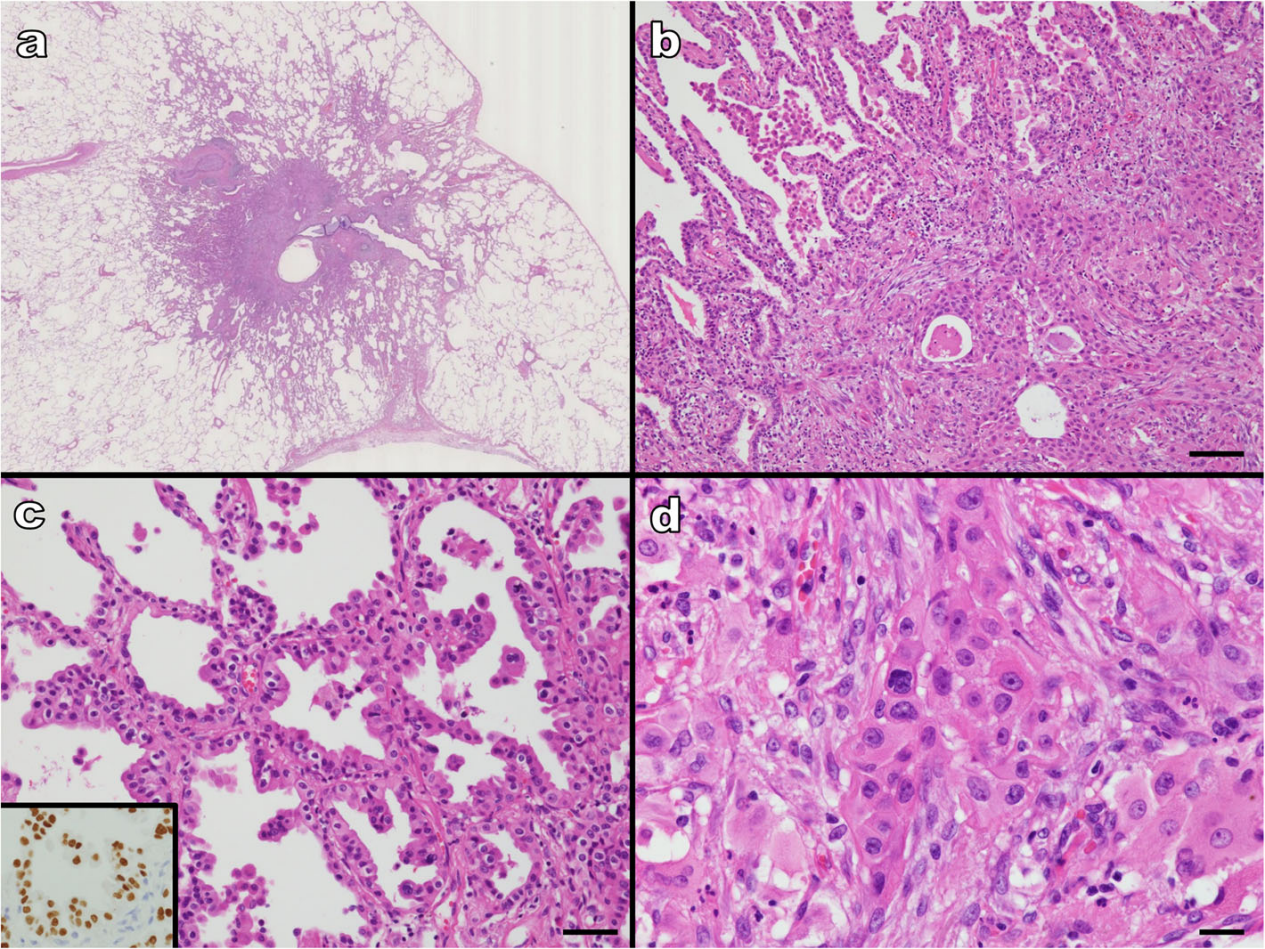
Tumor observation in the right lower lung lobectomy specimen. **a** Low magnification (× 0.5) imaging. A tumor with a central solid area is observed in the lung parenchyma; **b** (× 100 magnification). The upper left is the adenocarcinoma component, and the lower right is the squamous carcinoma component seen in the solid portion (scale bar 100 μm); **c** The adenocarcinoma shows a lepidic growth pattern (scale bar 50 μm). TTF-1 immunostaining is positive (inset); **d** (× 400 magnification) showing the squamous cell carcinoma component. Proliferation of atypical cells with acidophilic cytoplasm and intercellular bridges are observed (scale bar 20 μm)

**Fig. 4:**
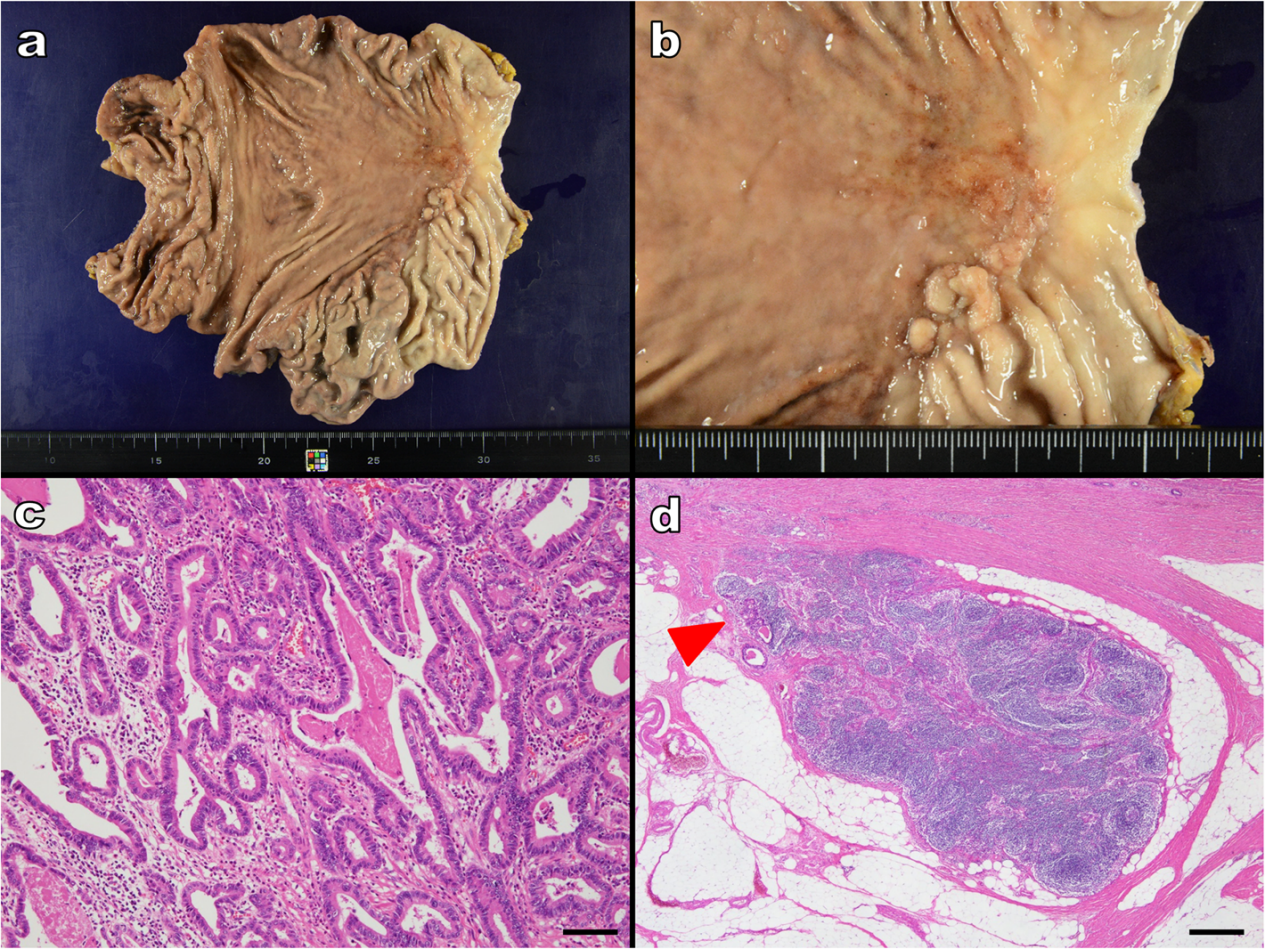
Recurrent gastric tumor. **a, b** Total gastrectomy specimen and enlarged photo of tumor. A flat, protruding lesion is present in the anastomotic region of the remaining portion of the stomach; **c** Histology of the recurrent tumor. Only growth of the adenocarcinomatous component is seen, without any sarcomatous components (scale bar 100 μm); **d** An extremely small lymph node metastasis was seen in 1 location of the total gastrectomy specimen (indicated by arrow; scale bar 500 μm)

Approximately 1 month following the post-gastrectomy, a chest CT showed a ground glass opacity in the inferior lobe of the patient’s right lung; after 6 months, the lung lesion grew, and therefore the patient underwent a right lower lobectomy. Histopathology of the resected tumor indicated a lepidic growth pattern rather than a gastric tumor metastasis. Further examination revealed that the tumor was a primary lung adenosquamous carcinoma with a well-differentiated lung adenocarcinoma component, positive for thyroid transcription factor 1 (TTF-1) on immunohistochemical staining, that was admixed with an SCC component (Fig. 3a-d). Fourteen months after the segmental gastrectomy, a flat, protruding lesion appeared in the anastomotic region of the patient’s remaining stomach portion. This lesion was identified as a local recurrence, and a total gastrectomy was carried out (Fig. 4a, b). Examination of the resected specimen from the local recurrence showed the growth of only the adenocarcinomatous component, without a sarcomatous component (Fig. 4c). The recurrent tumor extended into the subserosa, and we observed 1 very small site of lymph node metastasis (Fig. 4d). The patient started adjuvant chemotherapy by TS-1, and at the time of writing this report, the patient had survived for 3 months after the total gastrectomy. Although he had been diagnosed with three independent cancers, he did not undergo further evaluations for inherited cancer syndrome.

## Discussion

In the present case, the gastric carcinosarcoma was detected at an early stage, and both the carcinomatous and the sarcomatous components exhibited various types of differentiation. The carcinomatous component included an AFP-producing gastric carcinoma and areas that showed neuroendocrine differentiation, whereas the sarcomatous component included chondrosarcomatous, leiomyosarcomatous, and rhabdomyosarcomatous regions.

In most cases, however, gastric carcinosarcomas are discovered at advanced stages. There are reports of early-stage cases of gastric carcinosarcomas, but a report by Kuroda et al. showed that only 4 cases with tumor invasion limited to the submucosa were reported until 2017 [5]. To our knowledge, the present case is the 5th to be reported for this particular tumor stage. Previously, 1 of the patients with a gastric carcinosarcoma died of liver cirrhosis and hepatic encephalopathy [8]; however, there have been no reports of later local tumor recurrence despite the invasion being limited to the submucosa. Gastric carcinosarcoma is a neoplasm with a high rate of mortality and local recurrence. As this case illustrates, even in cases of early-stage gastric carcinosarcoma, it is necessary to watch for later recurrence and metastasis after surgical treatment.

In the present case, both the carcinomatous and sarcomatous components showed multi-type differentiation as described earlier. In general, for the histological findings of gastric carcinosarcoma, the carcinomatous and sarcomatous components are predominantly adenocarcinomatous and undifferentiated sarcoma, respectively. Of the 76 cases of gastric carcinosarcoma found in a literature review by Nie et al., 4 were adenosquamous carcinomas, and 7 were neuroendocrine carcinomas [3]. Reports of cases that include an AFP-producing gastric carcinoma component are limited to 2 cases from Japan [9, 10]. An evaluation of the sarcomatous component showed that sarcomas in 8 out of 76 cases contained at least 2 ectopic component types, and 2 cases contained 3 ectopic component types [3]. In the present case, we observed a unique histology, including more independently differentiated regions than in those reported in the literature. Immunohistochemistry was extremely useful in identifying these differentiations.

The etiopathogenetic mechanism of gastric carcinosarcoma remains unclear, but two hypotheses have been proposed thus far [5, 11]. The first is the bi-clonal origin hypothesis, which holds that the carcinosarcoma originates from 2 different tumor cell clones, which collide. The second is the monoclonal origin hypothesis – a view that a single-source stem cell differentiates into both a carcinoma and a sarcoma. The tumor in our patient contained diversely differentiated portions despite its early stage, tumor invasion limited to the submucosa, and the below-average size. Furthermore, with regard to germ cell multipotency, the germ cell-like cells positive for SALL4 – an immunohistochemical marker for germ cell tumors [12] – constituted a part of the tumor. In the present case, these findings support the monoclonal origin hypothesis wherein a single clone differentiates into a variety of morphologies, rather than the possibility of multiple tumor components colliding to form the lesion.

This patient had multiple primary cancers, esophageal SCC and primary lung adenosquamous carcinoma developed independently; the latter carcinoma is a rare tumor in itself. The appearance of a combination of rare tumors is another peculiarity of this patient, and the possibility of a genetic abnormality predisposing to cancer cannot be excluded.

## Conclusions

This case demonstrated early-stage, multi-differentiated gastric carcinosarcoma. Early-stage lesions may recur sometimes, as in the present case, and due care is necessary to detect recurrence and metastasis after gastric carcinosarcoma resection. The various types of differentiation of tumoral tissue and a germ-cell-like cell population in the present case potentially validate the monoclonal origin hypothesis in carcinosarcoma.

## Data Availability

The dataset supporting the findings and conclusions of this case report is included within this article.

## Abbreviations

AFP: Alpha-fetoprotein
SALL4: Sal-like protein 4
SCC: Squamous cell carcinoma
CEA: Carcinoembryonic antigen
N/C: Nucleus to cytoplasm
TTF-1: Thyroid transcription factor 1
